# Pregnancy rates from natural and artificial cycles of women submitted to frozen embryo transfers: a metanalysis

**DOI:** 10.5935/1518-0557.20190018

**Published:** 2019

**Authors:** Karine Queiroz Poletto, Mayana de Pina Lobo, Melissa Giovanucci, Mário Silva Approbato, Eduardo Camelo de Castro

**Affiliations:** 1 Laboratorio de Reprodução Humana (LabRep) / FM / HC - Federal University of Goiás - Brazil; 2 Pontifical Catholic University of Goiás - Brazil

**Keywords:** endometrium, pregnancy rate, cryopreservation

## Abstract

**Objective:**

To compare pregnancy rates from natural and artificial cycles of women submitted to frozen embryo transfers.

**Methods:**

A systematic review was performed by PubMed search using the following algorithm: (endometrial [All Fields] AND preparation [All Fields]) AND (("freezing"[MeSH Terms] OR "freezing"[All Fields] OR "frozen"[All Fields]) AND thawed [All Fields]) and (natural cycles) AND (artificial cycle). Inclusion criteria: prospective and retrospective cohort studies. Exclusion criteria: use of hCG in the natural cycle, oocyte donors, and use of disused freezing techniques. Data were analyzed with the SPSS v.23 software and with a significance level of 5%. The meta-analysis was performed with RevMan 5.3 software. I² was calculated.

**Results:**

709 papers were retrieved. Five studies fulfilled the inclusion and exclusion criteria. From these studies, we analyzed 8,968 natural or artificial cycles. A contingency table compared the results of the natural and artificial cycles and the number of clinical pregnancies obtained in each selected paper. The I^2^ test resulted in high statistical heterogeneity (I^2^=77%). Studies by Morozov *et al*. (2007) and Zheng *et al*. (2015) obtained statistically significant results (*p*<0.03 and *p*<0.001): Morozov *et al*. (2007) found a higher pregnancy rate within natural cycles, and Zheng *et al*. (2015) found more positive outcomes when analyzing artificial cycles. The remaining selected studies did not show any statistical significance.

**Conclusion:**

There is insufficient scientific evidence to state that the artificial cycle yields better pregnancy rates than the natural cycle in women submitted to frozen embryo transfer. Limitations of this study include a small number of papers and heterogeneity among the studies.

## INTRODUCTION

Adequate endometrial hormonal preparation is fundamental for the success of frozen embryo transfers in vitro fertilization cycles (Harper, 1992). Endometrium preparation can be achieved in a natural cycle after spontaneous ovulation or after using exogenous estrogen and progesterone.

In the natural ovulatory cycle, the hormonal secretion of the ovaries controls endometrium development. It undergoes a series of foreseeable changes associated with follicular development, ovulation and corpus luteum (Psychoyos, 1973). In the artificial cycle, estrogen and progesterone supplementation is used to mimic the normal cycle. The goal is to achieve adequate endometrial thickness to receive the embryo (Paulson, 2011).

Cryopreservation enables embryo transfer after ovarian stimulation for oocyte collection. It makes it possible to avoid ovarian hyperstimulation syndrome and to plan the options for the moment of the transfer (Corbett *et al*., 2014). The frozen embryo transfer success is closely linked to the exact synchronization between endometrial maturation and embryo development, as well as the transfer technique used (Diedrich *et al*., 2007; Mains & Van Voorhis, 2010). Reviews comparing the several endometrial preparation techniques were inconclusive (Glujovsky *et al*., 2010; Groenewoud *et al*., 2013; Ghobara *et al*., 2017).

The objective is to compare pregnancy rates among natural and artificial cycles of women submitted to frozen embryo transfers.

## MATERIALS AND METHODS

A meta-analysis was performed following the Preferred Reporting Items for Systematic Reviews and Meta-Analyses (PRISMA) statements, with an active search in the PubMed database from 2016-2017. The following descriptors were used: "Cryopreservation", "Embryo Transfer", "Endometrium/Estrogens", "Female", "Fertility Agents", "Pregnancy", "Pregnancy Rate".

The search algorithm used in the PubMed platform was the following: (Endometrial [All Fields] AND preparation [All Fields]) AND (("freezing" [MeSH Terms] OR "freezing" [All Fields] OR "frozen" [All Fields]) AND thawed [All Fields]) and (natural cycles) AND (artificial cycle).

Two independent reviewers evaluated the available papers. In case of disagreement, a third reviewer was called upon for further assessment. The inclusion criteria were description of endometrial preparation regimens, prospective and retrospective cohort studies comparing artificial cycles and natural cycles. The exclusion criteria were studies that lack information about endometrial preparation regimens, those that have not compared natural and artificial cycles, and those that did not describe the clinical pregnancy rates. Studies using hCG in the natural cycle (natural cycle modified) and those using oocyte donors were excluded.

**Figure 1:**
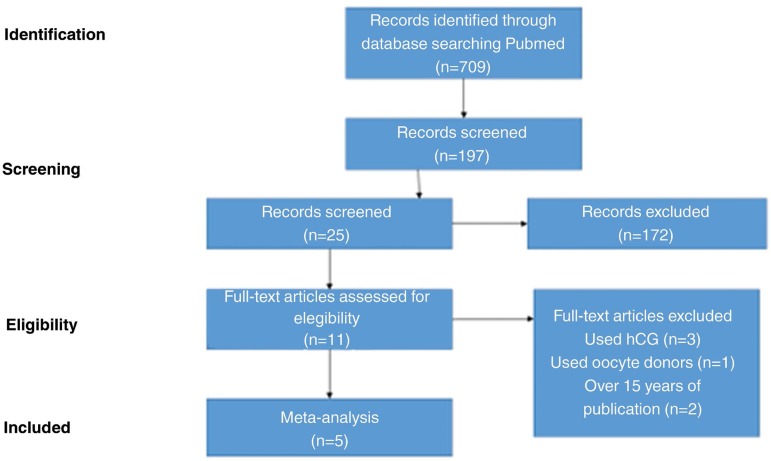
Flow diagram of the paper selection flowchart

In the pre-selection phase, we found 709 studies with the application of the descriptors. After using the inclusion and exclusion criteria, papers with over 15 years of publication were also excluded, because the most recent embryo freezing techniques are superior. A flow diagram demonstrating the process of paper selection and eligibility is demonstrated in Figure 1. Five retrospective cohort studies fulfilled the eligibility criteria and were selected.

The data was analyzed with the aid of the statistical package SPSS version 23, adopting a level of significance of 5% (*p*<0.05). The association between the type of gestation and the positive or negative outcome was performed based on the Pearson Chi-square test.

The meta-analysis was performed using the RevMan 5.3 software. The I^2^ was calculated for statistical heterogeneity. Values higher than 30% indicate heterogeneity. A random effect model was used in case of heterogeneity and a fixed-effect model in its absence. The Haenszel method was applied to estimate the pooled effects sizes. We used a significance level of *p* less than 0.05.

## RESULTS

A total of five studies were included with 8,968 natural or artificial cycles investigated. Table 1 depicts the variables extracted from the studies, including outcomes and protocols. This table includes the results of the natural and artificial cycles, and the number of clinical pregnancy rates obtained in each selected paper were compared.

**Table 1:** Selected studies and their protocols

Study	Design	Applied Protocols	Results	Conclusion
**Natural cycle cryo-thaw transfer may improve pregnancy outcomes.**	Retrospective Cohort	**Group 1: Artificial Cycle (n=174) **	**Pregnancies: **Group 1: 41	The use of hormonal supplementation showed a drop in pregnancy rates compared to the natural cycle.
		From day 2 of the cycle, estradiol started at 4 mg/day. If endometrial thickness at 13 days was greater than 8 mm, progesterone IM 50 mg/day was introduced and the transfer was done within 3 days.		
Morozov *et al*., 2007.		**Group 2: Natural Cycle (n = 68)**	Group 2: 25	
		Ovulation monitoring with US and serum LH. Transfer carried out 3 days after. Progesterone 800 mg per day was initiated.		
**Use of the natural cycle and vitrification thawed blastocyst transfer results in better in-vitro fertilization outcomes,**	Retrospective Cohort	**Group 1: Natural Cycle (n = 310)**	**Pregnancies:** Group 1: 130	There was no significant difference in pregnancy rates between the artificial and the natural cycles.
		Monitoring with US. Transfer performed approximately 5 days after ovulation. Initiated progesterone 600mg/day on the day of the transfer.		
		**Group 2:** **Natural Cycle + hCG (n=134)**	Group 2: 80	
Chang *et al*., 2011.		When the endometrium> 8mm and the dominant follicle> 20mm, ovulation was induced by 10,000 IU of hCG. (Results not assessed in the comparison of the present study)		
		Group 3 – Artificial Cycle (n=204)	Group 3: 85	
		From day 3 of the cycle, estradiol valerate started at 4 to 6 mg/day orally. If endometrial thickness at 14 days was not greater than 8 mm, the dose increased to 8 mg per day. If minimal thickness was reached, vaginal progesterone 600 mg per day was initiated.		
** Natural cycle is superior to hormone replacement therapy cycle for vitrificated-preserved frozen-thawed embryo transfer.**	Retrospective Cohort	**Group 1: Regular Natural Cycle (n = 380)**	**Pregnancies:** Group 1: 144	The results suggest that natural cycles are superior to hormonal cycles in certain circumstances and in a certain population of patients.
		Monitoring with US and serum LH. After ovulation progesterone, IM 40 mg was introduced. After 3 days, the transfer was made. **Group 2: Artificial Cycle (n=646)** From day 3 of the cycle estradiol valerate was introduced at 2 mg/day for 3 days, at 4 mg for 3 days, and 6 mg from the 10th day onwards. If the endometrium was then trilaminar with a thickness greater than 8 mm, progesterone IM 40 mg/day was started and the transfer was done in 3 days.		
Xiao *et al*., 2012.		**Group 2: Artificial Cycle (n=646)**	Group 2: 228	
		From day 3 of the cycle estradiol valerate was introduced at 2 mg/day for 3 days, at 4 mg for 3 days, and 6 mg from the 10th day onwards. If the endometrium was then trilaminar with a thickness greater than 8 mm, progesterone IM 40 mg/day was started and the transfer was done in 3 days.		
**Pregnancy loss after frozen-embryo transfer-a comparison of three protocols.**	Retrospective Cohort	**Group 1: Regular Natural Cycle (n = 1168):**	**Pregnancies: **Group 1: 248	A higher pregnancy rate was obtained in the artificial cycles, however, due to the increase of preclinical and clinical pregnancy loss, comparable clinical pregnancy and birth rates are reported for all three protocols.
		Monitoring with US and serum LH. Transfer was performed approximately 3-5 days after ovulation. It was then initiated 600 to 800 mg/day of vaginal progesterone, or Crione 90 mg twice daily on the day of transfer.		
		**Group 2: Natural Cycle + hCG (n=444):**	Group 2: 95	
Tomás *et al*., 2012.		After 10 days of spontaneous menstruation, when the endometrium was greater than 8mm and the dominant follicle reached 16-17mm in diameter, ovulation was induced by 5,000 IU of hCG, and embryo transferred 5 days later. (Results not assessed in the comparison of the present study) **Group 3: Artificial Cycle (n=2858) ** From day 1 of the cycle, estradiol started at 6 mg per day. If endometrial thickness at 10 days was greater than 7 mm, the transfer was made and vaginal progesterone 600 mg per day was started 4 days earlier.		
		**Group 3: Artificial Cycle (n=2858) **	Group 3: 691	
		From day 1 of the cycle, estradiol started at 6 mg per day. If endometrial thickness at 10 days was greater than 7 mm, the transfer was made and vaginal progesterone 600 mg per day was started 4 days earlier.		
**The artificial cycle method improves the pregnancy outcome in frozen-thawed embryo transfer: a retrospective cohort study**	Retrospective Cohort	**Group 1: Regular Natural Cycle (n = 654)**	**Pregnancies:** Group 1: 323	The study suggests superiority of the hormonal protocol.
		Monitoring with USG and serum LH and progesterone until the endometrial thickness is greater than 8 mm, after the transfer 60 mg of progesterone was initiated.		
Zheng *et al*., 2015		**Group 2: Artificial Cycle (n=2506)**	Group 2: 1469	
		From day 1 of the cycle, estradiol valerate of 2 mg per day was started for 4 days, 4 mg for another 4 days and 6 mg of 9th to 12th day thereafter. If endometrial> 8 mm, progesterone IM 40 mg/day was started and the transfer was done in 4 days. The luteal support was maintained with 60 mg of progesterone.		

The I^2^ test was applied to look for heterogeneity. Given the high statistical heterogeneity (I^2^=77%), we applied the random effects model to the meta-analysis.

Table 2 shows an association between the type of pregnancy and the outcome from each paper.

**Table 2:** Association between the type of pregnancy and the outcome of each article

Outcome	Pregnancy rate n (%)		Total	*x^2^*	p*
	Artificial	Natural			
**Chang *et al*., 2011**					
Negative	119 (58.3)	180 (58.1)	299 (58.2)	0.04	0.95
Positive	85 (41.7)	130 (41.9)	215 (41.8)		
Total	204	310	514		
**Morozov *et al*., 2007**					
Negative	133 (76.4)	43 (63.2)	176 (72.7)	**4.29**	**0.03**
Positive	41 (23.6)	25 (36.8)	66 (27.3)		
Total	174	68	242		
**Tomás *et al*., 2012**					
Negative	2167 (75.8)	920 (78.8)	3087 (76.7)	**4.02**	**0.05**
Positive	691 (24.2)	248 (21.2)	939 (23.3)		
Total	2858	1168	4026		
**Xiao *et al*., 2012**					
Negative	418 (64.7)	236 (62.1)	654 (63.7)	0.70	0.40
Positive	228 (35.3)	144 (37.9)	372 (36.3)		
Total	646	380	1026		
**Zheng *et al*., 2015**					
Negative	1037 (41.4)	331 (50.6)	1368 (43.3)	**18.00**	**<0.001**
Positive	1469 (58.6)	323 (49.4)	1792 (56.7)		
Total	2506	654	3160		

* Pearson's Chi-squared

**Figure 2:**
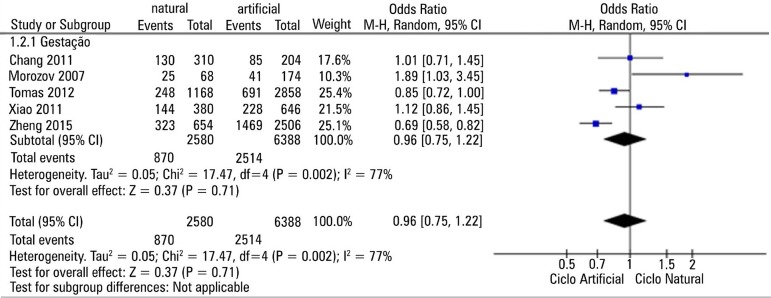
Artificial cycle x Natural cycle forest plot

Figure 2 presents a Forest Plot for the results obtained in the selected papers, with the comparison between artificial cycles versus natural cycles. We found no significant difference for the different types of stimulation protocol and pregnancy outcomes.

In the studies analyzed, only Morozov *et al*. (2007) and Zheng *et al*. (2015) found statistically significant results, being favorable for opposite results. While the former had a higher natural-cycle pregnancy rate, the latter had more positive outcomes with the artificial cycle. The other selected studies did not show statistical significance to support one cycle modality over the other.

## DISCUSSION

The estrogen administration doses and protocols varied in the interventions of the artificial cycles. Two studies used increasing doses of estrogen: Xiao *et al*. (2012) and Zheng *et al*. (2015). The other papers used constant estrogen doses. Studies suggest that the management of these doses may influence the final outcome, showing that using constant doses can result in a higher pregnancy rate (Madero *et al*., 2016). Studies were excluded where oocyte donations were used, since it is known that this is an independent factor that positively alters the results. Studies that select only specific types of infertility causes could further elucidate the issue.

Morozov *et al*. (2007) obtained significantly better results with the natural cycle and discuss the benefits of not using hormones, primarily in women with regular menstrual cycles. Some experimental models have shown that the implantation window would be smaller when the endometrium is exposed to higher levels of estrogen (Ma *et al*., 2003). The endometrial thickness of patients who had natural cycle gestation showed an average value close to 10 mm, corroborating that the average levels of the hormone present in the natural cycle would be sufficient to provide a developed endometrium, a predictor of success (Zhang *et al*., 2005), as well as a larger window for embryo implantation. Chang *et al*. (2011) argue that patients with normal ovarian function would benefit more from natural cycles since supraphysiological concentrations of estrogen would reduce the expression of the β3-subunit integrin and leukemia inhibitory factor (LIF) in the endometrium (Chen *et al*., 2008). The correlation between pregnancy rates and endometrial thickness was analyzed, and there was a statistically significant difference showing a good relationship between larger sizes and the success of the process.

Xiao *et al*. (2012) argue that a certain bias may have influenced their study, since it is retrospective and the patients had the method chosen according to their menstrual history - they were not randomized. They point out that in the artificial cycle there may be suppression of several hormones essential to maintain the pregnancy. Tomás *et al.* (2012) showed a higher number of pregnancies with artificial cycles, but also higher rates of abortion. The transfer of frozen embryos has higher loss rates when compared to the transfer of fresh embryos (Farr *et al*., 2007), without distinction of the protocol used. It is argued that there was a greater number of patients with polycystic ovary syndrome submitted to an artificial cycle, which may have influenced gestational loss. Similarly, Zheng *et al*. (2015) showed higher pregnancy rates in artificial cycles, but unlike the previous study, abortion rates were similar between the groups. The authors suggest that this happens not only because of the patient’s characteristics, but also because of the transfer method used in their study, in the blastocyst stage. They reported that hormonal supplementation is simpler and more flexible in relation to the moment of transfer, and it is better for women with irregular menstrual cycles.

This study presents some limitations that should be considered as they may jeopardize data generalization. These include the small number of studies retrieved and the high heterogeneity rate found among them. The lack of evaluation of additional variables such as patient’s age and embryo quality is also a limitation. However, we demonstrated that there is an important gap in the literature concerning this topic, as controversial results are found. In addition, more rigorous definitions and data access standardization should be considered in order to favor comparability among studies.

A method may be more favorable for a given population and such individualities could not be measured in the analysis we carried out. It is believed that other factors should be taken into account in choosing the method, such as costs, side effects and ease of use by the patient. Further randomized prospective studies are needed to define the best course of action to be followed, with greater efficiency, cost-effectiveness, safety and convenience.

## CONCLUSION

There is insufficient evidence to state that the artificial cycle offers better pregnancy rates than the natural cycle in women undergoing frozen embryo transfers. The results of this review should be carefully considered because of the small number of studies and the heterogeneity between the studies. It is suggested that more prospective studies comparing natural and artificial cycles are performed.
